# Utility of the combination of serum highly-sensitive C-reactive protein level at discharge and a risk index in predicting readmission for acute exacerbation of COPD*,**

**DOI:** 10.1590/S1806-37132014000500005

**Published:** 2014

**Authors:** Chun Chang, Hong Zhu, Ning Shen, Xiang Han, Yahong Chen, Bei He

**Affiliations:** Department of Respiratory Medicine, Peking University Third Hospital, Beijing, China; Department of Respiratory Medicine, Peking University Third Hospital, Beijing, China; Department of Respiratory Medicine, Peking University Third Hospital, Beijing, China; Department of Respiratory Medicine, Peking University Third Hospital, Beijing, China; Department of Respiratory Medicine, Peking University Third Hospital, Beijing, China

**Keywords:** Pulmonary disease, chronic obstructive/epidemiology, Acute disease, Acute-phase proteins, Hospitalization, Patient readmission, Inflammation

## Abstract

**OBJECTIVE:**

Frequent readmissions for acute exacerbations of COPD (AECOPD) are an independent risk factor for increased mortality and use of health-care resources. Disease severity and C-reactive protein (CRP) level are validated predictors of long-term prognosis in such patients. This study investigated the utility of combining serum CRP level with the Global Initiative for Chronic Obstructive Lung Disease (GOLD) exacerbation risk classification for predicting readmission for AECOPD.

**METHODS:**

This was a prospective observational study of consecutive patients hospitalized for AECOPD at Peking University Third Hospital, in Beijing, China. We assessed patient age; gender; smoking status and history (pack-years); lung function; AECOPD frequency during the last year; quality of life; GOLD risk category (A-D; D indicating the greatest risk); and serum level of high-sensitivity CRP at discharge (hsCRP-D).

**RESULTS:**

The final sample comprised 135 patients. Of those, 71 (52.6%) were readmitted at least once during the 12-month follow-up period. The median (interquartile) time to readmission was 78 days (42-178 days). Multivariate analysis revealed that serum hsCRP-D ≥ 3 mg/L and GOLD category D were independent predictors of readmission (hazard ratio = 3.486; 95% CI: 1.968-6.175; p < 0.001 and hazard ratio = 2.201; 95% CI: 1.342-3.610; p = 0.002, respectively). The ordering of the factor combinations by cumulative readmission risk, from highest to lowest, was as follows: hsCRP-D ≥ 3 mg/L and GOLD category D; hsCRP-D ≥ 3 mg/L and GOLD categories A-C; hsCRP-D < 3 mg/L and GOLD category D; hsCRP-D < 3 mg/L and GOLD categories A-C.

**CONCLUSIONS:**

Serum hsCRP-D and GOLD classification are independent predictors of readmission for AECOPD, and their predictive value increases when they are used in combination.

## Introduction

An acute exacerbation of COPD (AECOPD) is characterized by a worsening of respiratory symptoms beyond the normal day-to-day variation, requiring a change in medication.^(^ 1 ^)^ Severe AECOPDs require hospital admission and are responsible for up to 70% of COPD-related health care costs.^(^ 2 ^)^ After the index AECOPD, patients are at increased risk of readmission.^(^ 3 ^-^ 6 ^)^ Frequent readmissions due to AECOPD represent an independent risk factor for increased mortality.^(^ 4 ^)^

Proposed risk factors for readmission following hospitalization for AECOPD include aspects reflecting the underlying COPD severity, such as functional limitation and poor health-related quality of life.^(^ 7 ^)^ Because COPD is a systemic disease, multidimensional parameters such as the **B**ody mass index, airflow **O**bstruction, **D**yspnea, and **E**xercise capacity (BODE) index might be superior to FEV_1_ at reflecting COPD severity.^(^ 8 ^)^ Although the BODE index can be useful for predicting the need for hospitalization due to COPD,^(^ 9 ^)^ patients with impaired mobility are unable to perform the required six-minute walk test. The Global Initiative for Chronic Obstructive Lung Disease (GOLD) guidelines propose the combined assessment of symptoms, quality of life, spirometry measurements, and history of AECOPDs.^(^ 1 ^)^

One marker of systemic inflammation that is related to COPD prognosis is the serum level of C-reactive protein (CRP).^(^ 10 ^-^ 12 ^)^ Increased systemic inflammation during recovery from an AECOPD is associated with recurrence within 50 days.^(^ 10 ^)^ Whether serum CRP is a predictor of readmission for AECOPD during longer-term follow-up has yet to be determined.

Here, we investigated whether the GOLD disease severity classification and serum CRP level at discharge are predictors of readmission for AECOPD. We also attempted to determine whether the combination of the two constitutes a better predictor of readmission for AECOPD than does either used in isolation. 

## Methods

### Patients

This was a prospective, observational study of consecutive patients admitted to Peking University Third Hospital, a tertiary care center in Beijing, China, for AECOPD between 1 April of 2010 and 30 September of 2011. For patients admitted more than once during the study period, only the first admission was considered. The diagnosis of COPD was established by post-bronchodilator spirometry, in accordance with the GOLD guidelines.^(^ 1 ^)^ We defined AECOPD as acute, sustained worsening of the condition of a patient from a stable state to a level of severity that exceeded the normal day-to-day variation, thus necessitating a change in medication.^(^ 1 ^)^ Patients with a history of other respiratory illnesses, such as acute asthma, pulmonary tuberculosis, sleep apnea syndrome, bronchiectasis, or interstitial lung disease, were excluded, as were those for whom a respiratory physician or radiologist identified consolidation (i.e., pneumonia) on a chest X-ray, those not surviving the hospitalization period, and those hospitalized for reasons other than AECOPD. The study protocol was approved by the Research Ethics Committee of Peking University Third Hospital, Beijing (Approval no. IRB00001052-07095), and all participating patients gave written informed consent.

### Clinical variables

At admission, details were collected regarding preadmission COPD management. Spirometry values in the 6 months prior to study inclusion (when the COPD was stable) were obtained from patient records. Additional clinical and demographic data, described below, were obtained 8 weeks after hospital discharge, when the COPD was clinically stable. Disease duration was defined as the total duration of symptoms. Chest X-ray and electrocardiography were used in order to identify cor pulmonale, based on national criteria.^(^ 13 ^)^ Active smoking was defined as smoking within the past 6 months. All comorbidities were noted. Dyspnea was assessed using the modified Medical Research Council (mMRC) dyspnea scale.^(^ 14 ^)^ Patients completed the COPD Assessment Test (CAT), a questionnaire based on the symptoms experienced on that day. Based on their body mass index (BMI), calculated as weight in kilograms divided by height in meters squared (kg/m^2)^, patients were stratified into two groups: underweight (BMI < 20 kg/m^2)^; and normal-weight/overweight (BMI ≥ 20 kg/m^2)^. The self-reported number of AECOPDs during the last year, which is known to correlate well with the number of AECOPDs recorded on symptom diary cards,^(^ 15 ^)^ was taken as the frequency of AECOPDs.

### Classification of patients into GOLD categories

The GOLD classification stratifies patient risk first on the basis of symptoms, using the degree of dyspnea (mMRC score 0-1 vs. ≥ 2) or health status (CAT score < 10 vs. ≥ 10), into two low-symptom categories (A and C) and two high-symptom categories (B and D). The risk of AECOPD is assessed on the basis of the FEV_1_, calculated as a percentage of the predicted value (FEV_1_%: < 50% vs. ≥ 50%), or the number of AECOPDs in the last year (0-1 vs. ≥ 2), whichever is higher, and is used in order to stratify patients into two low-risk categories (A and B) and two high-risk categories (C and D).^(^ 1 ^)^ Therefore, category A indicates fewer symptoms and less risk; category B indicates more symptoms and less risk; category C indicates fewer symptoms and more risk; and category D indicates more symptoms and more risk.

### Blood sampling

Peripheral venous blood samples (7 mL) were collected from patients at admission (prior to treatment) and at discharge. After centrifugation at 6,716 × *g* for 10 min at 4°C, the plasma was separated and stored at −80°C for subsequent analysis.

### Determination of serum high-sensitivity CRP levels

The serum level of high-sensitivity CRP (hsCRP) was measured at discharge by a latex agglutination test in an automated chemistry immuno-analyzer (AU5400; Olympus, Tokyo, Japan) with a detection limit of 0.1 mg/L. Patients were stratified by hsCRP level: > 3 mg/L and ≤ 3 mg/L. The 3-mg/L cut-off value has been shown to be a determinant of the long-term prognosis.^(^ 10 ^-^ 12 ^)^

### Therapeutic strategy

In accordance with the GOLD guideline recommendations,^(^ 1 ^)^ hospitalized patients were treated with inhaled (nebulized) albuterol, ipratropium bromide, and budesonide, as well as with intravenous prednisolone (30-40 mg/day). After 4 days of intravenous prednisolone therapy, patients were switched to oral prednisolone, on a 10-14 day tapering schedule. If bacterial infection was suspected (on the basis of patient-reported sputum purulence), antibiotic therapy was initiated and was adjusted depending on antimicrobial susceptibilities, if known.

Mechanical ventilation (non-invasive, whenever possible) was instituted for conditions such as respiratory arrest, decreased level of consciousness, and elevated PaCO_2_ despite maximal pharmacological treatment. Decisions regarding admission or transfer to the ICU were made by the hospital staff. Cases of stable COPD were managed in accordance with the GOLD guidelines.^(^ 1 ^)^

### Follow-up

Patients were followed from the day of discharge until readmission or through August of 2012 if readmission did not occur. The primary outcome measure was time to readmission for AECOPD.

In monthly telephone interviews, patients were monitored to document the occurrence of AECOPDs and hospitalizations and completed a short questionnaire to assess any changes in respiratory symptoms and medical interventions during the past month. Patients were encouraged to report to their attending physicians whenever they experienced symptom worsening. An event-based AECOPD was confirmed if patients experienced worsening of at least one key symptom, plus a change in at least one of three medications (antibiotics, corticosteroids, or bronchodilators). The end of an AECOPD episode was defined as an improvement in symptoms (to their pre-AECOPD status or not) and symptom stabilization for at least 3 days. To distinguish relapse (symptom fluctuation during the same episode) from recurrence, readmissions within 14 days of the previous discharge were excluded from the final analysis. The need for hospitalization was determined in accordance with the GOLD guidelines.^(^ 1 ^)^

### Statistical analysis

Continuous variables are presented as median (interquartile range), and categorical variables are presented as absolute numbers and percentages. Descriptive statistics for the primary end points were determined by generating Kaplan-Meier curves of the time-to-event data. Univariate analysis of potential risk factors for the primary end points was performed with a log-rank test.

The time from discharge to first readmission for an AECOPD was used as the outcome variable in a Cox proportional hazards model. In the univariate analysis, significant predictors were entered in a stepwise fashion into a Cox proportional hazards model, in order to test the independent effect of each candidate risk factor.

Statistical analyses were performed using the Statistical Package for the Social Sciences, version 13.0 (SPSS Inc., Chicago, IL, USA). Values of p < 0.05 were considered statistically significant.

## Results

A total of 191 eligible patients were initially recruited. Of those, 14 (7.3%) did not survive to complete the recruitment process and 13 (6.7%) died during follow-up without having been readmitted for AECOPD; those patients were excluded from the analysis of risk factors for AECOPD readmission. An additional 7 patients (3.6%) were lost to follow-up, and 22 patients (11.4%) were readmitted within 14 days of discharge, therefore being excluded from the final analysis to distinguish between relapse and recurrence. Consequently, 135 patients were included in the final analysis, and their characteristics are shown in Table 1.

**Table 1:** Patient characteristics at baseline.

Characteristic	(n = 135)
Male gender, n (%)	119 (88.1)
Age (years), median (range)	66 (60-74)
Current smoker, n (%)	44 (32.6)
Pack years, median (range)	15 (11-27)
Duration of COPD (years), median (range)	9 (4-23)
Cor pulmonale, n (%)	49 (36.3)
FEV_1_ (% predicted), median (range)	47 (43-55)
CAT score, median (range)	16 (7-23)
mMRC score, median (range)	2 (1-3)
Number of AECOPDs in the last year, median (range)	2 (1-3)
GOLD category	
A, n (%)	24 (17.8)
B, n (%)	22 (16.3)
C, n (%)	22 (16.3)
D, n (%)	67 (49.6)
BMI (kg/m^2^), median (range)	22.5 (18.7-26.5)
Comorbidities	
Arterial hypertension, n (%)	41 (30.4)
Ischemic heart disease, n (%)	28 (20.7)
Diabetes, n (%)	20 (14.8)
Congestive heart failure, n (%)	15 (11.1)
Renal disease, n (%)	9 (6.7)
Hepatic disease, n (%)	6 (4.4)
Cerebrovascular disease, n (%)	6 (4.4)
Pre-admission therapy for COPD	
Home oxygen therapy, n (%)	32 (23.7)
Corticosteroid therapy, n (%)	75 (55.6)
Inhaled corticosteroid^a^, n (%)	64 (85.3)
Oral corticosteroid^b^, n (%)	11 (14.7)

CAT, COPD Assessment Test; mMRC, modified Medical Research Council (dyspnea scale); AECOPD: acute exacerbation of COPD; and BMI, body-mass index. aFluticasone propionate at = 500 µg/day for more than 12 months. bAny oral corticosteroid used on a regular basis (treatment for more than 3 months with prednisone at 7.5 mg/day, or equivalent).

In the study sample as a whole, the median serum hsCRP level was significantly lower on the day of discharge than at admission (3.2 mg/L [2.0-5.6 mg/L] vs. 7.8 mg/L [6.6-12.2 mg/L]; Z = −9.319, p < 0.001).

Patients were followed for a median of 284 days (76-408 days). There were 71 patients (52.6%) who were readmitted at least once during the follow-up period. The median time to readmission was 78 days (42-178 days). 

In the univariate analysis, a serum hsCRP level ≥ 3 mg/L at discharge, advanced age, ≥ 2 AECOPDs in the last year, FEV_1_% < 50%, a CAT score ≥ 10, and being in GOLD category D were all associated with a significantly increased risk of AECOPD readmission (Table 2).

**Table 2:** Univariate analysis of risk factors for readmission for acute exacerbation of COPD.

Factor	No readmission	Readmission	p
	(n = 64)	(n = 71)	
Serum hsCRP at discharge			0.000
< 3 mg/L, n (%)	45 (70.3)	16 (22.5)	
≥ 3 mg/L, n (%)	19 (29.7)	55 (77.5)	
GOLD category			0.000
A-C, n (%)	44 (68.8)	24 (33.8)	
D, n (%)	20 (31.3)	47 (66.2)	
Age (years), median (range)	64 (58-70)	68 (62-75)	0.017
FEV_1_ (% of predicted)			0.002
≥ 50%, n (%)	39 (60.9)	24 (33.8)	
< 50%, n (%)	25 (39.1)	47 (66.2)	
Number of AECOPDs in the last year			0.000
< 2, n (%)	47 (73.4)	40 (56.3)	
≥ 2, n (%)	17 (26.6)	31 (43.4)	
CAT score			0.003
< 10, n (%)	30 (46.9)	16 (22.5)	
≥ 10, n (%)	34 (53.1)	55 (77.5)	
BMI			0.494
< 20 kg/m^2^, n (%)	19 (29.7)	25 (35.2)	
≥ 20 kg/m^2^, n (%)	45 (70.3)	46 (64.8)	
mMRC score			0.385
≥2, n (%)	39 (60.9)	38 (53.5)	
<2, n (%)	25 (39.1)	33 (46.5)	
Pre-admission therapy for COPD			
Home oxygen therapy			0.379
Yes, n (%)	13 (20.3)	19 (26.8)	
No, n (%)	51 (79.7)	52 (73.2)	
Corticosteroid therapy			
Inhaled corticosteroid^a^, n (%)	30 (46.9)	34 (47.9)	0.906
Oral corticosteroid^b^, n (%)	3 (4.7)	8 (11.3)	0.163
Cor pulmonale			0.061
Yes, n (%)	18 (28.1)	31 (43.4)	
No, n (%)	46 (71.9)	40 (56.3)	
Comorbidities			
Arterial hypertension, n (%)	18 (28.1)	23 (32.4)	0.59
Ischemic heart disease, n (%)	11 (17.2)	17 (23.9)	0.334
Diabetes, n (%)	12 (18.8)	8 (11.3)	0.222
Congestive heart failure, n (%)	7 (10.9)	8 (11.3)	0.951
Renal disease, n (%)	4 (6.3)	5 (7.0)	1.000
Current smoking			0.248
Yes, n (%)	24 (37.5)	20 (28.2)	
No, n (%)	40 (62.5)	51 (71.8)	

hsCRP: high-sensitivity C-reactive protein; AECOPD: acute exacerbation of COPD; CAT, COPD Assessment Test; BMI, body-mass index; and mMRC, modified Medical Research Council (dyspnea scale). aFluticasone propionate at = 500 µg/day for more than 12 months. bAny oral corticosteroid used on a regular basis (treatment for more than 3 months with prednisone at 7.5 mg/day, or equivalent).

**Figure 1:**
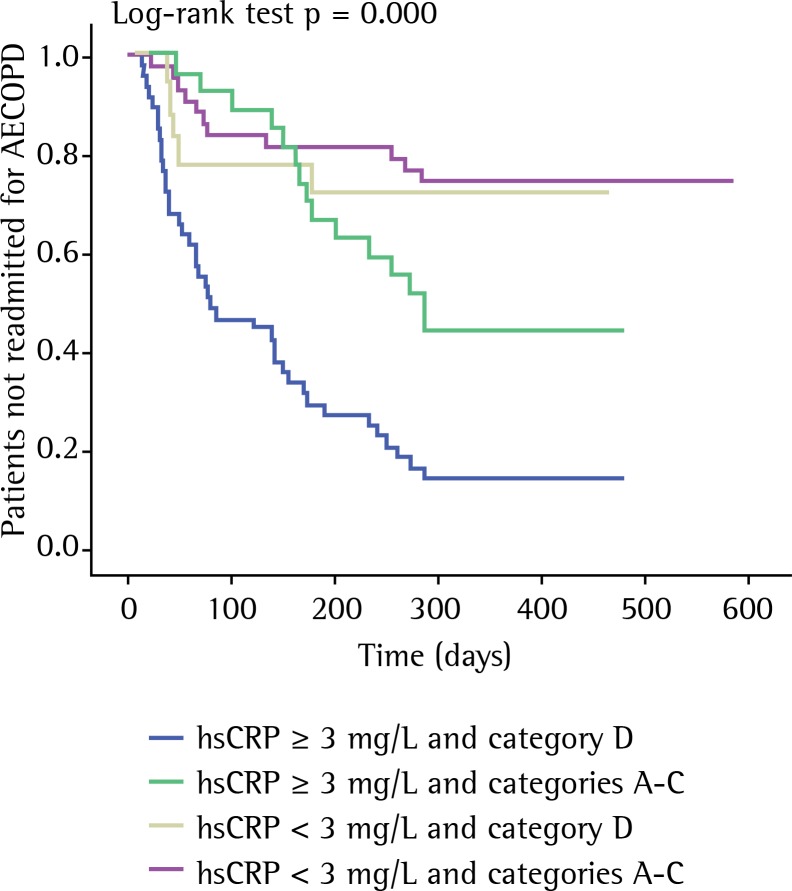
Kaplan-Meier curves showing readmission for acute exacerbation of COPD (AECOPD) during the 12-month follow-up period after the index AECOPD. The curves for readmission are presented for the four subgroups of the patient cohort, categorized on the basis of the patient serum level of high-sensitivity C-reactive protein (hsCRP) at discharge and the Global Initiative for Chronic Obstructive Lung Disease (GOLD) risk category. The order of the groups, ranked from highest to lowest readmission rate (p < 0.001), was as follows: serum hsCRP = 3 mg/L and GOLD category D; serum hsCRP = 3 mg/L and GOLD categories A-C; serum hsCRP < 3 mg/L and GOLD category D; and serum hsCRP < 3 mg/L and GOLD categories A-C.

In the multivariate analysis, we used two models that differed in their consideration of serum hsCRP level: model 1, in which serum hsCRP was treated as a continuous variable; and model 2, in which it was classified into two categories (≥ 3 mg/L and < 3 mg/L). The results of the multivariate analysis showed that the serum hsCRP level at discharge (considered as either a continuous or categorical variable) and being in GOLD category D both remained as independent predictors of a higher risk of readmission for AECOPD (Table 3). In patients discharged after being treated for an AECOPD, the combination of a serum hsCRP level ≥ 3 mg/L at discharge and being in GOLD category D predicted a higher risk of readmission for AECOPD than did any other combination of the (presence or absence) two factors (Figure 1).

**Table 3:** Cox proportional hazards models of risk factors for readmission for acute exacerbation of COPD.

Model	p	OR	95% CI
Variable			
1			
Serum hsCRP level at discharge (continuous)	0.000	1.173	1.084-1.269
GOLD category D vs. GOLD categories A-C	0.001	2.330	1.413-3.843
2			
Serum hsCRP level at discharge (≥ 3 mg/L vs. < 3 mg/L)	0.000	3.486	1.968-6.175
GOLD category D vs. GOLD categories A-C	0.002	2.201	1.342-3.610

hsCRP: high-sensitivity C-reactive protein; and GOLD: Global Initiative for Chronic Obstructive Lung Disease.

## Discussion

The main findings of this prospective study of patients hospitalized for AECOPD are that being in GOLD category D and having an elevated serum hsCRP level at discharge are independent predictors of the risk of readmission for AECOPD, and that the combination of serum hsCRP level ≥3 mg/L at discharge and being in GOLD category D disease severity is predictive of a much higher risk of readmission than the presence of either factor alone. We propose that combined analysis of these risk factors will allow for better stratification of the risk of readmission for AECOPD.

The high AECOPD readmission rate observed in our study (52.6%), which is similar to those reported previously,^(^ 3 ^,^ 16 ^)^ demonstrates the weight of the socioeconomic burden of COPD. Because recurrent hospital admissions for AECOPD constitute an independent risk factor for increased mortality,^(^ 4 ^)^ it is clinically important to identify patients at increased risk of readmission, which can allow early implementation of preventive strategies.

Indices related to the severity of the underlying COPD are independent predictors of readmission, including the number of prior admissions for AECOPD,^(^ 3 ^,^ 5 ^,^ 6 ^,^ 17 ^-^ 20 ^)^ FEV_1_,^(^ 1 ^,^ 3 ^,^ 5 ^,^ 18 ^,^ 21 ^)^ and quality of life. ^(^ 22 ^)^ There is growing recognition that COPD is a multidimensional disease.^(^ 1 ^,^ 23 ^,^ 24 ^)^ Multidimensional grading systems seem to offer better insight into outcomes such as survival and the need for hospitalization.^(^ 9 ^,^ 25 ^,^ 26 ^)^ Although the BODE index is the most widely studied multidimensional score,^(^ 8 ^)^ we used the GOLD classification in order to avoid any bias related to the exclusion of patients with impaired mobility who would be unable to perform the six-minute walk test. The GOLD classification does not require sophisticated technology and can be applied in any clinical situation or location.^(^ 1 ^)^ To our knowledge, this is the first study to show an association between the GOLD classification and the risk of readmission for AECOPD.

Serum CRP level is considered a valid biomarker of systemic inflammation in patients with COPD, as well as a predictor of poor COPD prognosis. ^(^ 10 ^-^ 12 ^)^ A serum CRP level > 3 mg/L has been shown to be an independent predictor of future COPD-related hospitalization and death.^(^ 12 ^)^ In addition, all-cause mortality and the annual incidence of moderate/severe AECOPDs are higher in patients with elevated systemic inflammatory biomarkers.^(^ 27 ^)^ Persistently elevated serum CRP during recovery from an AECOPD is associated with AECOPD recurrence within 50 days.^(^ 10 ^)^ In our study, serum hsCRP during the recovery period was significantly associated with readmission due to AECOPD, after adjustment for age, FEV_1_%, CAT score, frequency of AECOPDs in the last year, and GOLD category.

A previous study found that treatment with fluticasone, with or without salmeterol, was not associated with significant effects on inflammatory biomarkers (CRP or IL-6) in patients with COPD. ^(^ 28 ^)^ Further interventional studies are needed in order to determine whether therapies targeted at patients with a high hsCRP level at discharge can reduce or prevent readmissions for AECOPD, thereby decreasing morbidity, mortality, and health care costs.

In the present study, we stratified patients hospitalized for AECOPD by readmission risk, from highest to lowest (p < 0.001), as follows: hsCRP ≥ 3 mg/L and being in GOLD category D; hsCRP ≥ 3 mg/L and being in GOLD categories A-C; hsCRP < 3 mg/L and being in GOLD category D; hsCRP < 3 mg/L and being in GOLD categories A-C. This is consistent with a previous report identifying the combination of low serum CRP and a low BODE score as a better predictor of survival than either parameter alone.^(^ 29 ^)^ Our study thus indicates that combining a systemic inflammatory marker with multidimensional disease severity grading is superior at predicting readmission for AECOPD than is either factor alone. 

The results of the present study underscore the importance of patient follow-up (e.g., through routine determination of the serum hsCRP level at discharge). The components of the GOLD grading system can be easily acquired in many health care settings and could be integrated into the follow-up of discharged patients at no additional cost. Hence, the combination of the GOLD classification and serum hsCRP level at discharge could be used routinely in clinical practice to risk stratify patients hospitalized for AECOPD.

One strength of the present study is its prospective design. The loss rate during follow-up was very low. An obvious limitation of our study is that it involved only a modest number of patients treated at a single facility, and our findings therefore cannot be the generalized without further confirmation in studies involving larger numbers of patients recruited from multiple facilities. Another limitation is that some readmissions during follow-up might have represented relapses of previous AECOPDs rather than new AECOPDs. However, we attempted to minimize that problem by not considering readmissions occurring within 14 days of the previous discharge. Yet another potential limitation is that the CAT and mMRC scores were acquired during stable convalescence (8 weeks after discharge), and we therefore cannot exclude the possibility that the treatments administered during hospitalization had prolonged effects on those scores. However, that is unlikely to have interfered with the interpretation of our results, because the AECOPD treatment was standardized. In addition, a previous study reported a median time for the CAT score to return to baseline of 11 days (4.5-17 days).^(^ 15 ^)^ Another factor that might have influenced the risk of readmission was patient adherence to treatment, although regular monthly follow-up is likely to have improved adherence. Furthermore, certain markers thought to reflect AECOPD severity were not evaluated. However, those markers are more important predictors of in-hospital mortality than of mortality after discharge or of readmission.^(^ 7 ^)^

In summary, our data confirm the supposition that serum hsCRP level at discharge and GOLD category are independent predictors of readmission for AECOPD. A serum hsCRP level ≥ 3 mg/L at discharge and being in GOLD category D indicated the highest risk of readmission. Additional cohort studies involving larger sample sizes will determine the validity of our results.
