# Supplementary material for: Utility of the combination of serum highly-sensitive C-reactive protein level at discharge and a risk index in predicting readmission for acute exacerbation of COPD
Source: J Bras Pneumol. 2014 Sep-Oct;40(5):495–503. doi: 10.1590/S1806-37132014000500005 (PMC4263330; doi:10.1590/S1806-37132014000500005)
Supplement: Translation

# Translation: Utilidade de se combinar o nível sérico de proteína C reativa de alta sensibilidade no momento da alta com um índice de risco para prever a reinternação por exacerbação aguda da DPOC*

**Authors:** Chun Chang, Hong Zhu, Ning Shen, Xiang Han, Yahong Chen, Bei He

**Affiliations:** Terceiro Hospital da Universidade de Pequim, Pequim, China; Terceiro Hospital da Universidade de Pequim, Pequim, China; Terceiro Hospital da Universidade de Pequim, Pequim, China; Terceiro Hospital da Universidade de Pequim, Pequim, China; Terceiro Hospital da Universidade de Pequim, Pequim, China; Terceiro Hospital da Universidade de Pequim, Pequim, China

**Keywords:** Doença pulmonar obstrutiva crônica/epidemiologia, Doença aguda, Hospitalização, Readmissão do paciente, Inflamação

## Abstract

**OBJETIVO:**

Reinternações frequentes por exacerbações agudas da DPOC (EADPOC) são um fator de risco independente para maior mortalidade e uso de recursos de saúde. A gravidade da doença e o nível de proteína C reativa (PCR) são preditores validados do prognóstico em longo prazo para tais pacientes. Investigamos a utilidade da combinação do nível sérico de PCR com a classificação de risco de exacerbação da *Global Initiative for Chronic Obstructive Lung Disease* (GOLD) para predizer a reinternação por EADPOC.

**MÉTODOS:**

Estudo observacional prospectivo de pacientes consecutivos hospitalizados por EADPOC no *Peking University Third Hospital*, in Pequim, China. Avaliamos a idade; gênero, história e carga tabágicas (anos-maço), função pulmonar, frequência de EADPOC no último ano; qualidade de vida; categoria de risco GOLD (A-D, D indicando maior risco); e nível sérico de PCR de alta sensibilidade na alta (PCRas-A).

**RESULTADOS:**

A amostra final consistiu em 135 pacientes. Desses, 71 (52,6%) foram reinternados ao menos uma vez durante o período de seguimento de 12 meses. A mediana (intervalo interquartílico) do tempo de reinternação foi de 78 dias (42-178 dias). A análise multivariada revelou que PCRas-A sérico ≥ 3 mg/L e categoria GOLD D foram preditores independentes de reinternação (razão de risco = 3,486; IC95%: 1,968-6,175; p < 0,001 e razão de risco = 2,201; IC95%: 1,342-3,610; p = 0,002, respectivamente). A ordem das combinações dos fatores por risco cumulativo de readmissão, da maior para a menor foi a seguinte: PCRas-A ≥ 3 mg/L e categoria GOLD D; PCRas-A ≥ 3 mg/L e categorias GOLD A-C; PCRas-A < 3 mg/L e categoria GOLD D; e PCRas-A < 3 mg/L e categorias GOLD A-C.

**CONCLUSÕES:**

O nível sérico de PCRas-A e a classificação GOLD são preditores independentes de reinternação por EADPOC, e seu valor preditivo aumenta quanto usados conjuntamente.

## Introdução

Uma exacerbação aguda da DPOCL (EADPOC) é caracterizada por um agravamento dos sintomas respiratórios que vai além da variação diária normal e que requer uma mudança na medicação.^(^ 1 ^)^ Graves EADPOC requerem internação hospitalar e são responsáveis por até 70% dos custos de assistência à saúde relacionados com a DPOC. ^(^ 2 ^)^ Após uma EADPOC, o risco de reinternação aumenta.^(^ 3 ^-^ 6 ^)^ Reinternações frequentes por motivo de EADPOC são um fator de risco independente de aumento da mortalidade.^(^ 4 ^)^

Dentre os possíveis fatores de risco de reinternação após uma internação por EADPOC estão aspectos que refletem a gravidade subjacente da DPOC, tais como limitação funcional e má qualidade de vida relacionada à saúde.^(^ 7 ^)^ Como a DPOC é uma doença sistêmica, é possível que parâmetros multidimensionais como o índice B*ody mass index*, *airflow *O*bstruction*, D*yspnea*, *and *E*xercise capacity* (BODE) reflitam a gravidade da DPOC melhor que o VEF_1_.^(^ 8 ^)^ Embora o índice BODE possa ser útil para prever a necessidade de hospitalização por DPOC,^(^ 9 ^)^ pacientes com mobilidade reduzida não são capazes de realizar o teste de caminhada de seis minutos necessário. As diretrizes da *Global Initiative for Chronic Obstructive Lung Disease* (GOLD) propõem que se avaliem conjuntamente sintomas, qualidade de vida, medidas espirométricas e história de EADPOC.^(^ 1 ^)^

O nível sérico de proteína C reativa (PCR) é um marcador de inflamação sistêmica que está relacionado com o prognóstico da DPOC.^(^ 10 ^-^ 12 ^)^ O aumento da inflamação sistêmica durante o período em que se recupera de uma EADPOC está relacionado com recorrência dentro de 50 dias.^(^ 10 ^)^ Ainda não se sabe se a PCR sérica é um preditor de reinternação por EADPOC durante o acompanhamento em longo prazo. 

Aqui, investigamos se a classificação da gravidade da doença da GOLD e o nível sérico de PCR no momento da alta são preditores de reinternação por EADPOC. Tentamos também determinar se conjuntamente constituem um preditor de reinternação por EADPOC melhor do que um ou outro separadamente. 

## Métodos

### Pacientes

Trata-se de um estudo observacional prospectivo de pacientes consecutivos que, entre 1º de abril de 2010 e 30 de setembro de 2011, foram admitidos no Terceiro Hospital da Universidade de Pequim - um centro de atendimento terciário em Pequim, na China - por motivo de EADPOC. Para pacientes internados mais de uma vez durante o estudo, considerou-se apenas a primeira internação. O diagnóstico de DPOC foi estabelecido por meio de espirometria pós-broncodilatador, de acordo com as diretrizes da GOLD.^(^ 1 ^)^ Considerou-se EADPOC o agravamento agudo e continuado do estado de um paciente, de um estado estável para um grau de gravidade maior que a variação diária normal, sendo, portanto, necessário mudar a medicação.^(^ 1 ^)^ Foram excluídos pacientes com história de outras doenças respiratórias, tais como asma aguda, tuberculose pulmonar, síndrome da apneia do sono, bronquiectasia e doença pulmonar intersticial, bem como aqueles nos quais um médico ou radiologista especialista no sistema respiratório identificou consolidação (isto é, pneumonia) em radiografia de tórax, aqueles que não sobreviveram ao período de internação e aqueles hospitalizados por outros motivos que não EADPOC. O protocolo do estudo foi aprovado pelo Comitê de Ética em Pesquisa do Terceiro Hospital da Universidade de Pequim (Aprovação nº IRB00001052-07095), e todos os pacientes participantes assinaram um termo de consentimento livre e esclarecido. 

### Variáveis clínicas

No momento da admissão hospitalar, foram coletados os detalhes a respeito de como a DPOC fora tratada antes da admissão. Os valores espirométricos nos 6 meses anteriores à inclusão no estudo (quando a DPOC estava estável) foram extraídos dos prontuários médicos dos pacientes. Dados clínicos e demográficos adicionais, descritos a seguir, foram obtidos 8 semanas após a alta hospitalar, quando a DPOC estava clinicamente estável. A duração total dos sintomas foi considerada a duração da doença. A radiografia de tórax e a eletrocardiografia foram usadas para identificar cor pulmonale, com base em critérios nacionais.^(^ 13 ^)^ Tabagismo nos últimos 6 meses foi considerado tabagismo ativo. Todas as comorbidades foram anotadas. A dispneia foi avaliada por meio da versão modificada da escala de dispneia do *Medical Research Council* (mMRC).^(^ 14 ^)^ Os pacientes completaram o *COPD Assessment Test* (CAT, Teste de Avaliação da DPOC), um questionário baseado nos sintomas apresentados no dia. Com base em seu índice de massa corporal (IMC) - o peso em quilogramas dividido pela estatura em metros quadrados (kg/m^2)^ - os pacientes foram estratificados em dois grupos: peso baixo (IMC < 20 kg/m^2)^ e peso normal/sobrepeso (IMC ≥ 20 kg/m^2)^. O número de EADPOC durante o ano anterior segundo os próprios pacientes, que é conhecido por apresentar boa correlação com o número de EADPOC registrado em diário de sintomas,^(^ 15 ^)^ foi tomado como a frequência de EADPOC. 

### Classificação dos pacientes em categorias da GOLD

A classificação da GOLD estratifica o risco primeiro com base em sintomas, por meio do grau de dispneia (pontuação na mMRC = 0-1 vs. ≥ 2) ou do estado de saúde (pontuação no CAT < 10 vs. ≥ 10), em duas categorias de poucos sintomas (A e C) e duas de muitos sintomas (B e D). O risco de EADPOC é avaliado com base no VEF_1_ em porcentagem do valor previsto (VEF_1_%: < 50% vs. ≥ 50%) ou no número de EADPOC no ano anterior (0-1 vs. ≥ 2) - o que for maior - e é usado para estratificar pacientes em duas categorias de pouco risco (A e B) e duas de muito risco (C e D).^(^ 1 ^)^ Portanto, a categoria A indica menos sintomas e menos risco; a categoria B indica mais sintomas e menos risco; a categoria C indica menos sintomas e mais risco e a categoria D indica mais sintomas e mais risco. 

### Coleta de amostras de sangue

Amostras de sangue venoso periférico (7 mL) foram extraídas dos pacientes no momento da admissão (antes do tratamento) e no momento da alta. Após a centrifugação a 6.716 × *g* durante 10 min a 4°C, o plasma foi separado e armazenado a −80°C para análise subsequente. 

### Determinação dos níveis séricos de PCR de alta sensibilidade

O nível sérico de PCR de alta sensibilidade (PCRas) foi medido no momento da alta por meio de um teste de aglutinação do látex em um imunoanalisador químico automático (AU5400; Olympus, Tóquio, Japão) com limite de detecção de 0,1 mg/L. Os pacientes foram estratificados de acordo com o nível de PCRas: > 3 mg/L e ≤ 3 mg/L. Demonstrou-se que o valor de corte de 3 mg/L é uma determinante do prognóstico em longo prazo.^(^ 10 ^-^ 12 ^)^

### Estratégia terapêutica

Em conformidade com as diretrizes da GOLD,^(^ 1 ^)^ os pacientes hospitalizados foram tratados com salbutamol inalado (nebulizado), brometo de ipratrópio, budesonida e prednisolona intravenosa (30-40 mg/dia). Após 4 dias de terapia com prednisolona intravenosa, os pacientes passaram a receber prednisolona oral, com redução gradual a cada 10-14 dias. Se houvesse suspeita de infecção bacteriana (baseada na presença, segundo os próprios pacientes, de escarro purulento), iniciava-se a antibioticoterapia, que era ajustada de acordo com a suscetibilidade a antimicrobianos, caso esta fosse conhecida. 

Instituiu-se a ventilação mecânica (não invasiva, sempre que possível) em casos de parada respiratória, diminuição do nível de consciência e PaCO_2_ elevada não obstante o tratamento farmacológico máximo. As decisões a respeito da admissão de pacientes ou de sua transferência para a UTI foram tomadas pela equipe do hospital. Casos de DPOC estável foram tratados de acordo com as diretrizes da GOLD.^(^ 1 ^)^

### Acompanhamento

Os pacientes foram acompanhados desde o dia da alta até a reinternação ou até o fim de agosto de 2012 quando não houve reinternação. O desfecho primário foi o tempo até a reinternação por EADPOC. 

Em entrevistas telefônicas mensais, os pacientes foram monitorados a fim de documentar a ocorrência de EADPOC e hospitalizações e completaram um breve questionário para avaliar quaisquer mudanças nos sintomas respiratórios e intervenções médicas durante o mês anterior. Os pacientes foram encorajados a se apresentar a seus médicos assistentes sempre que apresentassem piora dos sintomas. Confirmava-se EADPOC baseada em eventos caso os pacientes experimentassem o agravamento de pelo menos um dos sintomas principais, além de mudança em pelo menos um de três medicamentos (antibióticos, corticosteroides ou broncodilatadores). Uma melhoria dos sintomas (retorno ao estado pré-EADPOC ou não) e a estabilização dos sintomas durante pelo menos 3 dias foram consideradas o fim de um episódio de EADPOC. Para distinguir recidiva (flutuação de sintomas durante o mesmo episódio) de recorrência, reinternações dentro de 14 dias após a alta anterior foram excluídas da análise final. A necessidade de internação foi determinada em conformidade com as diretrizes da GOLD.^(^ 1 ^)^

### Análise estatística

As variáveis contínuas são apresentadas na forma de mediana (intervalo interquartil), e as variáveis categóricas são apresentadas na forma de números absolutos e porcentagens. As estatísticas descritivas para os desfechos primários foram determinadas por meio de curvas de Kaplan-Meier para os dados relativos ao tempo até determinado evento. A análise univariada dos possíveis fatores de risco dos desfechos primários foi realizada por meio de um teste de *log-rank*. 

O tempo decorrido entre a alta e a primeira reinternação por EADPOC foi usado como variável de desfecho em um modelo de riscos proporcionais de Cox. Na análise univariada, os preditores significativos foram introduzidos gradualmente em um modelo de riscos proporcionais de Cox a fim de testar o efeito independente de cada fator de risco candidato. 

As análises estatísticas foram realizadas por meio do programa *Statistical Package for the Social Sciences*, versão 13.0 (SPSS Inc., Chicago, IL, EUA). Valores de p < 0,05 foram considerados estatisticamente significantes.

## Resultados

Foram inicialmente recrutados 191 pacientes aptos a participar do estudo. Desses 191 pacientes, 14 (7,3%) não sobreviveram para completar o processo de recrutamento e 13 (6,7%) morreram durante o acompanhamento sem terem sido reinternados por EADPOC; esses pacientes foram excluídos da análise de fatores de risco de reinternação por EADPOC. Mais 7 pacientes (3,6%) foram perdidos durante o período de acompanhamento, e 22 pacientes (11,4%) foram reinternados em até 14 dias após a alta, sendo, portanto, excluídos da análise final para distinguir recaída de recorrência. Consequentemente, 135 pacientes foram incluídos na análise final, e suas características são apresentadas na Tabela 1. 

**Tabela 1:** Características basais dos pacientes.

Característica	(n = 135)
Gênero masculino, n (%)	119 (88,1)
Idade (anos), mediana (variação)	66 (60-74)
Fumante, n (%)	44 (32,6)
Anos-maço, mediana (variação)	15 (11-27)
Duração da DPOC (anos), mediana (variação)	9 (4-23)
Cor pulmonale, n (%)	49 (36,3)
VEF_1_ (% do previsto), mediana (variação)	47 (43-55)
Pontuação no CAT, mediana (variação)	16 (7-23)
Pontuação na mMRC, mediana (variação)	2 (1-3)
Número de EADPOC no último ano, mediana (variação)	2 (1-3)
Categoria GOLD	
A, n (%)	24 (17,8)
B, n (%)	22 (16,3)
C, n (%)	22 (16,3)
D, n (%)	67 (49,6)
IMC (kg/m^2^), mediana (variação)	22,5 (18,7-26,5)
Comorbidades	
Hipertensão arterial, n (%)	41 (30,4)
Cardiopatia isquêmica, n (%)	28 (20,7)
Diabetes, n (%)	20 (14,8)
Insuficiência cardíaca congestiva, n (%)	15 (11,1)
Doença renal, n (%)	9 (6,7)
Doença hepática, n (%)	6 (4,4)
Doença cerebrovascular, n (%)	6 (4,4)
Tratamento da DPOC antes da internação	
Oxigenoterapia domiciliar, n (%)	32 (23,7)
Corticoterapia, n (%)	75 (55,6)
Corticosteroide inalatório^a^, n (%)	64 (85,3)
Corticosteroide oral^b^, n (%)	11 (14,7)

CAT: COPD Assessment Test (Teste de Avaliação da DPOC); mMRC: versão modificada da escala de dispneia do Medical Research Council; EADPOC: exacerbação aguda da DPOC; e IMC: índice de massa corporal

a Propionato de fluticasona a = 500 µg/dia durante mais de 12 meses

b Qualquer corticosteroide oral usado regularmente (tratamento durante mais de 3 meses com prednisona a 7,5 mg/dia ou equivalente).

Na amostra como um todo, a mediana do nível sérico de PCRas foi significativamente menor no dia da alta do que no momento da internação [3,2 mg/L (2,0-5,6 mg/L) vs. 7,8 mg/L (6,6-12,2 mg/L); Z = −9,319, p < 0,001]. 

Os pacientes foram acompanhados durante uma mediana de 284 dias (76-408 dias). Houve 71 pacientes (52,6%) que foram reinternados pelo menos uma vez durante o período de acompanhamento. A mediana do tempo até a reinternação foi de 78 dias (42-178 dias). 

Na análise univariada, um nível sérico de PCRas ≥ 3 mg/L no momento da alta, a idade avançada, ≥ 2 EADPOC no ano anterior, VEF_1_% < 50%, uma pontuação ≥ 10 no CAT e estar na categoria GOLD D relacionaram-se com um aumento significativo do risco de reinternação por EADPOC (Tabela 2). 

**Tabela 2:** Análise univariada de fatores de risco de reinternação por exacerbação aguda da DPOC.

Fator	Sem reinternação	Reinternação	p
	(n = 64)	(n = 71)	
PCRas sérica no momento da alta			0,000
< 3 mg/L, n (%)	45 (70,3)	16 (22,5)	
≥ 3 mg/L, n (%)	19 (29,7)	55 (77,5)	
Categoria GOLD			0,000
A-C, n (%)	44 (68,8)	24 (33,8)	
D, n (%)	20 (31,3)	47 (66,2)	
Idade (anos), mediana (variação)	64 (58-70)	68 (62-75)	0,017
VEF_1_ (% do previsto)			0,002
≥ 50%, n (%)	39 (60,9)	24 (33,8)	
< 50%, n (%)	25 (39,1)	47 (66,2)	
Número de EADPOC no último ano			0,000
< 2, n (%)	47 (73,4)	40 (56,3)	
≥ 2, n (%)	17 (26,6)	31 (43,4)	
Pontuação no CAT			0,003
< 10, n (%)	30 (46,9)	16 (22,5)	
≥ 10, n (%)	34 (53,1)	55 (77,5)	
IMC			0,494
< 20 kg/m^2^, n (%)	19 (29,7)	25 (35,2)	
≥ 20 kg/m^2^, n (%)	45 (70,3)	46 (64,8)	
Pontuação na mMRC			0,385
≥2, n (%)	39 (60,9)	38 (53,5)	
<2, n (%)	25 (39,1)	33 (46,5)	
Tratamento da DPOC antes da internação			
Oxigenoterapia domiciliar			0,379
Sim, n (%)	13 (20,3)	19 (26,8)	
Não, n (%)	51 (79,7)	52 (73,2)	
Corticoterapia			
Corticosteroide inalatório^a^, n (%)	30 (46,9)	34 (47,9)	0,906
Corticosteroide oral^b^, n (%)	3 (4,7)	8 (11,3)	0,163
Cor pulmonale			0,061
Sim, n (%)	18 (28,1)	31 (43,4)	
Não, n (%)	46 (71,9)	40 (56,3)	
Comorbidades			
Hipertensão arterial, n (%)	18 (28,1)	23 (32,4)	0,59
Cardiopatia isquêmica, n (%)	11 (17,2)	17 (23,9)	0,334
Diabetes, n (%)	12 (18,8)	8 (11,3)	0,222
Insuficiência cardíaca congestiva, n (%)	7 (10,9)	8 (11,3)	0,951
Doença renal, n (%)	4 (6,3)	5 (7,0)	1,000
Tabagismo			0,248
Sim, n (%)	24 (37,5)	20 (28,2)	
Não, n (%)	40 (62,5)	51 (71,8)	

PCRas: proteína C reativa de alta sensibilidade; EADPOC: exacerbação aguda da DPOC; CAT: COPD Assessment Test (Teste de Avaliação da DPOC); IMC: índice de massa corporal; e mMRC: versão modificada da escala de dispneia do Medical Research Council

a Propionato de fluticasona a = 500 µg/dia durante mais de 12 meses

b Qualquer corticosteroide oral usado regularmente (tratamento durante mais de 3 meses com prednisona a 7,5 mg/dia ou equivalente).

Na análise multivariada, foram usados dois modelos que consideraram o nível sérico de PCRas de modo distinto: o modelo 1, em que a PCRas sérica foi tratada como uma variável contínua, e o modelo 2, em que a PCRas sérica foi classificada em duas categorias (≥ 3 mg/L e < 3 mg/L). Os resultados da análise multivariada mostraram que o nível sérico de PCRas no momento da alta (considerado uma variável contínua ou categórica) e estar na categoria GOLD D permaneceram como preditores independentes de maior risco de reinternação por EADPOC (Tabela 3). Nos pacientes que receberam alta após o tratamento de uma EADPOC, a combinação de nível sérico de PCRas ≥ 3 mg/L no momento da alta com categoria GOLD D previu um risco de reinternação por EADPOC maior do que qualquer outra combinação (da presença ou ausência) dos dois fatores (Figura 1). 

**Tabela 3:** Modelos de riscos proporcionais de Cox de fatores de risco de reinternação por exacerbação aguda da DPOC.

Modelo	p	OR	IC95%
Variável			
1			
Nível sérico de PCRas no momento da alta (contínua)	0,000	1,173	1,084-1,269
Categoria GOLD D vs. categoria GOLD A-C	0,001	2,330	1,413-3,843
2			
Nível sérico de PCRas no momento da alta (≥ 3 mg/L vs. < 3 mg/L)	0,000	3,486	1,968-6,175
Categoria GOLD D vs. categoria GOLD A-C	0,002	2,201	1,342-3,610

PCRas: proteína C reativa de alta sensibilidade; e GOLD: Global Initiative for Chronic Obstructive Lung Disease.

**Figura 1:** Curvas de Kaplan-Meier mostrando a reinternação por exacerbação aguda da DPOC (EADPOC) durante o período de 12 meses de acompanhamento após a primeira EADPOC. As curvas de reinternação são apresentadas para os quatro subgrupos da coorte de pacientes, categorizados em função do nível sérico de proteína C reativa de alta sensibilidade (PCRas) no momento da alta e da categoria de risco da Global Initiative for Chronic Obstructive Lung Disease (GOLD). A ordem dos grupos, da maior para a menor taxa de reinternação (p < 0,001), foi a seguinte: PCRas sérica = 3 mg/L e categoria GOLD D; PCRas sérica = 3 mg/L e categoria GOLD A-C; PCRas sérica < 3 mg/L e categoria GOLD D; PCRas sérica < 3 mg/L e categoria GOLD A-C.

## Discussão

Os principais achados deste estudo prospectivo de pacientes hospitalizados por EADPOC são que estar na categoria GOLD D e ter elevado nível sérico de PCRas no momento da alta são preditores independentes do risco de reinternação por EADPOC, e que a combinação de nível sérico de PCRas ≥ 3 mg/L no momento da alta com categoria GOLD D de gravidade da doença prevê um risco de reinternação muito maior do que o faz a presença de apenas um dos dois fatores isoladamente. Acreditamos que a análise combinada desses fatores de risco permitirá que se estratifique melhor o risco de reinternação por EADPOC. 

A alta taxa de reinternação por EADPOC observada em nosso estudo (52,6%), semelhante às relatadas anteriormente,^(^ 3 ^,^ 16 ^)^ demonstra o ônus socioeconômico da DPOC. Como internações hospitalares recorrentes em virtude de EADPOC constituem um fator de risco independente de aumento da mortalidade,^(^ 4 ^)^ é clinicamente importante identificar pacientes nos quais o risco de reinternação é maior, pois isso possibilita a implantação precoce de estratégias preventivas. 

Índices relacionados com a gravidade da DPOC subjacente são preditores independentes de reinternação, dentre os quais estão o número de internações prévias em virtude de EADPOC,^(^ 3 ^,^ 5 ^,^ 6 ^,^ 17 ^- 20)^ o VEF_1_^(^ 1 ^,^ 3 ^,^ 5 ^,^ 18 ^,^ 21 ^)^ e a qualidade de vida.^(^ 22 ^)^ Há crescente reconhecimento de que a DPOC é uma doença multidimensional. ^(^ 1 ^,^ 23 ^,^ 24 ^)^ Sistemas de classificação multidimensional parecem propiciar uma compreensão mais clara de desfechos como a sobrevida e a necessidade de hospitalização. ^(^ 9 ^,^ 25 ^,^ 26 ^)^ Embora o índice BODE seja o sistema de pontuação multidimensional mais estudado,^(^ 8 ^)^ usamos a classificação da GOLD a fim de evitar qualquer viés relacionado com a exclusão de pacientes com mobilidade reduzida que seriam incapazes de realizar o teste de caminhada de seis minutos. A classificação da GOLD não requer tecnologia sofisticada e pode ser aplicada em qualquer situação clínica ou local.^(^ 1 ^)^ Até onde sabemos, este é o primeiro estudo a mostrar uma associação entre a classificação da GOLD e o risco de reinternação por EADPOC. 

O nível sérico de PCR é considerado um biomarcador válido de inflamação sistêmica em pacientes com DPOC, bem como um preditor de mau prognóstico da DPOC.^(^ 10 ^-^ 12 ^)^ Demonstrou-se que um nível sérico de PCR > 3 mg/L é um preditor independente de futura hospitalização e morte por DPOC.^(^ 12 ^)^ Além disso, a mortalidade por todas as causas e a incidência anual de EADPOC moderada/grave são maiores em pacientes com biomarcadores inflamatórios sistêmicos elevados. ^(^ 27 ^)^ A PCR sérica persistentemente elevada durante o período em que se recupera de uma EADPOC relaciona-se com recorrência de EADPOC dentro de 50 dias.^(^ 10 ^)^ Em nosso estudo, a PCRas sérica durante o período de recuperação relacionou-se significativamente com reinternação por EADPOC, após ajuste de idade, VEF_1_%, pontuação no CAT, frequência de EADPOC no ano anterior e categoria GOLD. 

Um estudo anterior mostrou que o tratamento com fluticasona, com ou sem salmeterol, não se relacionou com efeitos significativos sobre biomarcadores inflamatórios (PCR ou IL-6) em pacientes com DPOC.^(^ 28 ^)^ Mais estudos de intervenção são necessários para determinar se terapias dirigidas a pacientes com alto nível de PCRas no momento da alta podem reduzir ou prevenir as reinternações por EADPOC, diminuindo assim a morbidade, a mortalidade e os custos da assistência à saúde. 

No presente estudo, pacientes hospitalizados por EADPOC foram estratificados de acordo com o risco de reinternação, do maior para o menor (p < 0,001), como se segue: PCRas ≥ 3 mg/L e categoria GOLD D; PCRas ≥ 3 mg/L e categoria GOLD A-C; PCRas < 3 mg L e categoria GOLD D; PCRas < 3 mg/L e categoria GOLD A-C. Isso está de acordo com um estudo anterior que identificou a combinação de baixa PCR sérica com baixo índice BODE como um preditor de sobrevida melhor do que qualquer um dos dois parâmetros isoladamente.^(^ 29 ^)^ Nosso estudo indica, portanto, que a combinação de um marcador inflamatório sistêmico com uma classificação multidimensional da gravidade da doença é superior a qualquer um dos dois fatores separadamente quanto à capacidade de prever reinternação por EADPOC. 

Os resultados do presente estudo ressaltam a importância de se acompanhar os pacientes (por exemplo, por meio da determinação rotineira do nível sérico de PCRas no momento da alta). Os componentes do sistema de classificação da GOLD podem ser facilmente obtidos em muitos cenários de assistência à saúde e poderiam ser integrados, sem nenhum custo adicional, ao acompanhamento de pacientes que receberam alta hospitalar. Assim, a combinação da classificação da GOLD com o nível sérico de PCRas no momento da alta poderia ser usada rotineiramente na prática clínica para estratificar os pacientes hospitalizados por EADPOC de acordo com o risco que correm. 

Um ponto forte do presente estudo é seu delineamento prospectivo. A taxa de perda durante o acompanhamento foi muito baixa. Uma limitação óbvia de nosso estudo é que ele envolveu um número modesto de pacientes, tratados em um único centro, e nossos achados não podem, portanto, ser generalizados sem a confirmação obtida por meio de estudos envolvendo um maior número de pacientes recrutados em diversos centros. Outra limitação é que algumas das reinternações ocorridas durante o acompanhamento podem ter representado recaídas de EADPOC anteriores, e não novas EADPOC. No entanto, tentamos reduzir ao máximo esse problema ao não considerar reinternações ocorridas dentro de 14 dias após a alta anterior. Outra possível limitação é que a pontuação no CAT e na mMRC foi obtida durante a convalescença estável (8 semanas após a alta), e, portanto, não se pode excluir a possibilidade de que os tratamentos administrados durante a internação tenham tido efeitos prolongados sobre essas pontuações. No entanto, é improvável que isso tenha interferido na interpretação de nossos resultados, pois o tratamento da EADPOC foi padronizado. Além disso, um estudo anterior mostrou uma mediana de 11 dias (4,5-17 dias) para que a pontuação no CAT retornasse à pontuação basal.^(^ 15 ^)^ Outro fator que pode ter influenciado o risco de reinternação foi a adesão dos pacientes ao tratamento, embora seja provável que o acompanhamento mensal regular tenha melhorado a adesão. Além disso, não foram avaliados certos marcadores que, segundo se acredita, refletem a gravidade da EADPOC. No entanto, esses marcadores são preditores mais importantes de mortalidade hospitalar do que de mortalidade após a alta ou de reinternação.^(^ 7 ^)^

Em suma, nossos dados confirmam a suposição de que o nível sérico de PCRas no momento da alta e a categoria GOLD são preditores independentes de reinternação por EADPOC. Apresentar nível sérico de PCRas ≥ 3 mg/L no momento da alta e estar na categoria GOLD D indicaram o maior risco de reinternação. Estudos de coorte adicionais com amostras maiores irão determinar a validade de nossos resultados.
